# Traumatic brain injury impact on normal hearing

**DOI:** 10.1016/j.cjtee.2025.05.006

**Published:** 2026-02-05

**Authors:** Fazle Kibria, Sudip Kumar Das

**Affiliations:** a Lovelace Biomedical Research Institute, Albuquerque, NM, 87108, USA; b Department of Otolaryngology and Ear, Nose, Throat Surgery, Kolkata Medical College and Hospital, Kolkata, 700073, India

**Keywords:** Traumatic brain injury, Wallerian degeneration, Cerebrospinal fluid, Auditory system, Hearing loss, Ear damage

## Abstract

Traumatic brain injury (TBI) incidents bring drastic health adversities in multiple systems of the body. The immediate impact on the head and central nervous system is promptly accelerated to the autonomous and peripheral nervous system. It causes an instant variability in intracranial pressure, heart rate, blood pressure, normal function of chemo/baro receptors, and sudden failure in normal communication. These effects promote long-term secondary health adversity, such as nerve schwannoma, gliosis, Wallerian degeneration, mitochondrial dysfunction, change in blood-brain barrier pathology, and impaired drainage of cerebrospinal fluid. The focal and diffuse character of TBI has a primary effect on the auditory system in terms of outer, middle, and inner ear damage. Auditory nerve dysfunction, central auditory miscommunication with the brain, intracanalicular vestibular schwannomas, lesions in cortical regions, hair cell damage, and spiral ganglion injury cause long-term secondary consequences on normal hearing function associated with TBI. In this present work, normal hearing impairment due to TBI-induced nerve damage, central-peripheral auditory dysfunction, long-term hearing loss, and associated other health impacts are highlighted.

## 1 Introduction

**Fig. 1:**
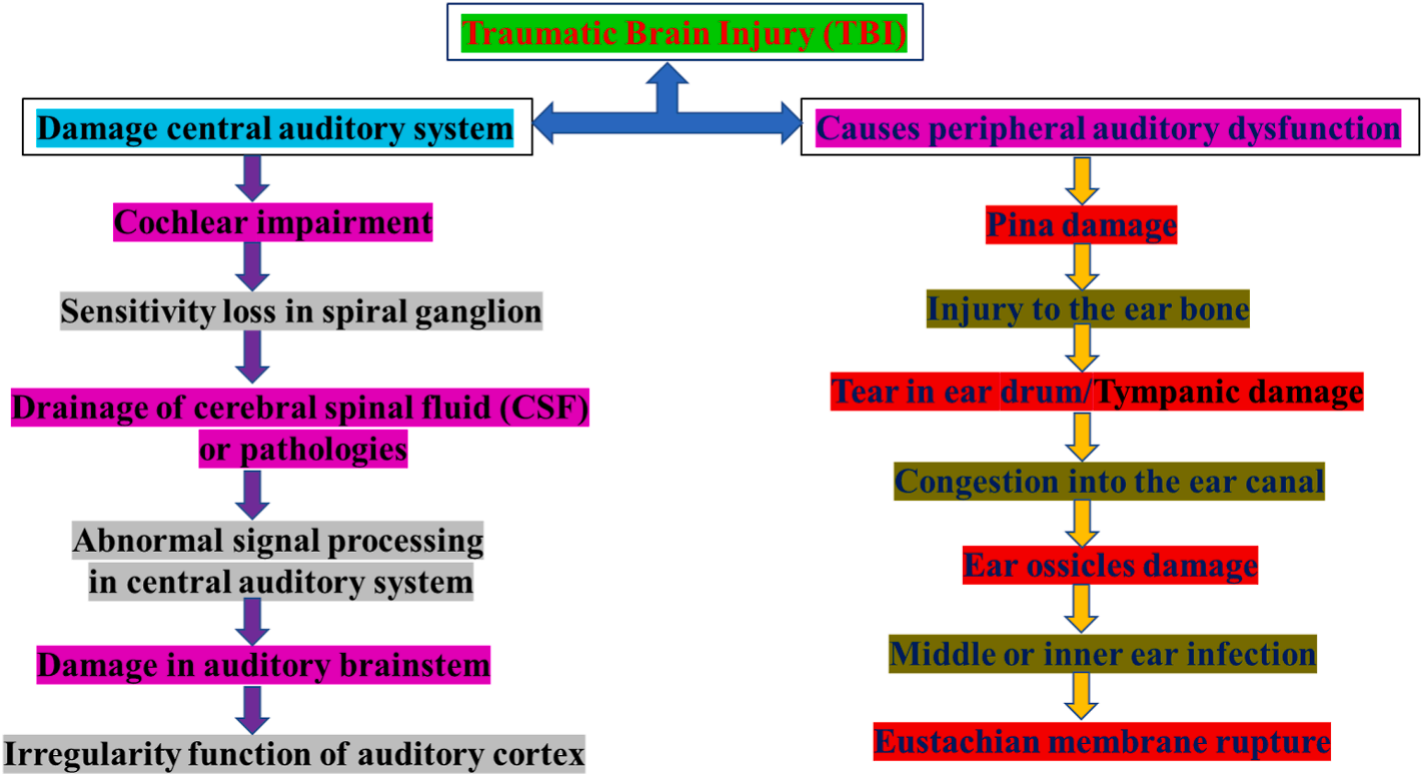
Traumatic brain injury impact on central and peripheral auditory function.

A traumatic brain injury (TBI) incident even once in a lifetime may introduce several health impacts to the victims, few are appearing immediately such as headache, unconsciousness, body part injury, drainage of cerebrospinal fluid (CSF), hemorrhage, cardiac dysfunction, etc. as a primary outcome of TBI and others, such as Alzheimer's disease, Parkinson's disease, dementia, dystonia, psycho-behavioral abnormality, problem in communication and auditory dysfunctions, are identified later in the life as a secondary post-TBI outcome maybe days or years after the actual scene. A recent literature cited that 0.9% to 58% of TBI victims suffer from hearing loss or any kind of auditory dysfunction.^1^ The statistical analysis based on the latest data showed that hearing complexities are 2 – 16 times higher in TBI patients than in individuals without a history of TBI.^2^ TBI is known as mild, moderate, and severe based on the severity, clinical abnormalities, and structural changes.^3^ A concussion is synonymous with mild TBI caused by a blunt, non-penetrating head impact demonstrating minimum clinical consequences. Severe is the highest level of TBI caused by car accidents, falls, and heavy assault on the head, leading to loss of consciousness for extended periods and lifelong adverse health impacts. Moderate TBI is between mild and severe, where the victim suffers unconsciousness for less than 7 days.^4^ All categories of TBI can cause short or long-term hearing impairment. However, which particular TBI causes maximum impact on auditory function is yet to be revealed. Focal and diffuse features of TBI impact may cause discernible adversities to various components of the auditory system, which are directly or indirectly maintaining normal hearing and auditory functions. The latest findings revealed that TBI may affect ear bones in the outer, middle, and inner ear; damage the auditory nerve; and impair cortical regions, which are associated with normal auditory input/output function and response processing.^5^ TBI severity on auditory function is not limited to a particular extent; it can be central auditory dysfunction, cochlear impairment, tympanic damage, causing difficulty with normal hearing speech in noise, hypo or hypersensitivity to sounds, loss of postural balance due to CSF drainage or pathologies, tinnitus, and partial or complete hearing loss.^6^ TBI impact introduces damage to the inner ear, spinal cord, central auditory system, auditory brainstem, and auditory cortex, which promotes hearing loss.^2^^,^^7^^,^^8^ Continuous research findings indicated that hair cell damage and sensitivity loss in the spiral ganglion are associated with TBI outcomes.^2^ The complex heterogeneous effect of TBI can cause auditory dysfunction like auditory response processing disorder, cochlear malfunction, neurodegeneration due to hair cell loss, tinnitus, and complex auditory processing, even in the absence of direct linear impact on the auditory system.^1^^,^^8^, ^9^, ^10^ Fig. 1 represents TBI-associated central and peripheral impairment of auditory function. The normal auditory function is mainly performed on receipt of incident sound energy at the external ear, which then transmits through the ear canal, 3 ear bones (malleus, incus, and stapes), oval window, eardrum, and at the tympanic membrane in terms of vibration. The oval window converts the vibrational energy into a fluid wave through the perilymph in the cochlea. The round window works as an outlet to maintain normal fluid pressure.^11^ The vibrational mechanism of sound propagation generates a directional flow and proportional pressure gradient following equation (1), within the perilymph, which activates the intact cochlear hair cells for auditory perception during the signal transfer process.

Equation 1 $$\frac{\partial^{2}P}{\partial x^{2}}=\frac{1}{c^{2}}\frac{\partial^{2}P}{\partial t^{2}}--------------$$

Where P is the acoustic pressure and c is the velocity of sound in the double derivative of the conventional equation above.

Any change in the pressure velocity gradient is an indication of ear impairment, which sometimes causes little discomfort, such as mild tinnitus. A severe TBI incident causes random drainage of CSF, which reduces the supply of perilymph to the scala tympani and alters the pressure gradient, leading to imbalance and audiometric dysfunction.^12^ The latest findings on the mouse model showed that repeated TBI introduced in chronic auditory dysfunction by damaging the spiral ganglion without affecting hair cells, which indicated systematic neuronal damage similar to neuronal death in the brain promoted by TBI.^2^ To understand the severity of hearing dysfunction, we may quote the words of the great Helen Keller “Blindness cuts us off from things, but deafness cuts us off from people”. Therefore, greater care should be taken to treat TBI-associated hearing problems as early as possible because there is evidence of auditory dysfunction after childhood TBI.^5^^,^^13^ Auditory hallucination was identified in a greater extent of the chronic TBI patients, nearly about 12% – 98%, which causes distress and further life complexities.^14^ However, post-TBI hearing impairment is sometimes ignored due to a lack of treatment options, unconsciousness of the patients, and little or no immediate comprehensible effect after the TBI incident. The main objective of this work is to highlight exclusive facts of TBI-associated auditory dysfunction in light of auditory neuronal impairment and hearing loss in other health conditions.

## 2 Methods

The search platforms Google Scholar and PubMed were utilized for systematic literature hunting with the keywords TBI, brain injury, hearing dysfunction, auditory dysfunction after brain injury, hearing loss in TBI, and the impact of TBI-associated hearing loss. The obtained results were reviewed according to the subject of interest. The literature review extensively included manuscripts written in the English language only. Information was extracted and summarized for review. This work is intended to refine the published work on the topic and exclude a meta-analysis or methodological review of the literature.

## 3 The vestibulocochlear nerve in hearing function

The primary major nerve that is responsible for hearing-balance function in mammals is the vestibulocochlear. The name vestibulocochlear is a combination of the vestibular and cochlear nerves. It's the 8th cranial nerve, where the vestibular nerve helps to maintain balance by detecting the motion of the head and body. On the other hand, the cochlear nerve controls acoustic signals through the hearing system by detecting the source of sound and providing necessary responses. Both vestibular and cochlear nerves originate from the brainstem. After their origin, the nerves merge to form the vestibulocochlear nerve located in the posterior cranial fossa in the petrous part of the temporal bone, which is again split in the inner ear to perform their specific job.

**Fig. 2:**
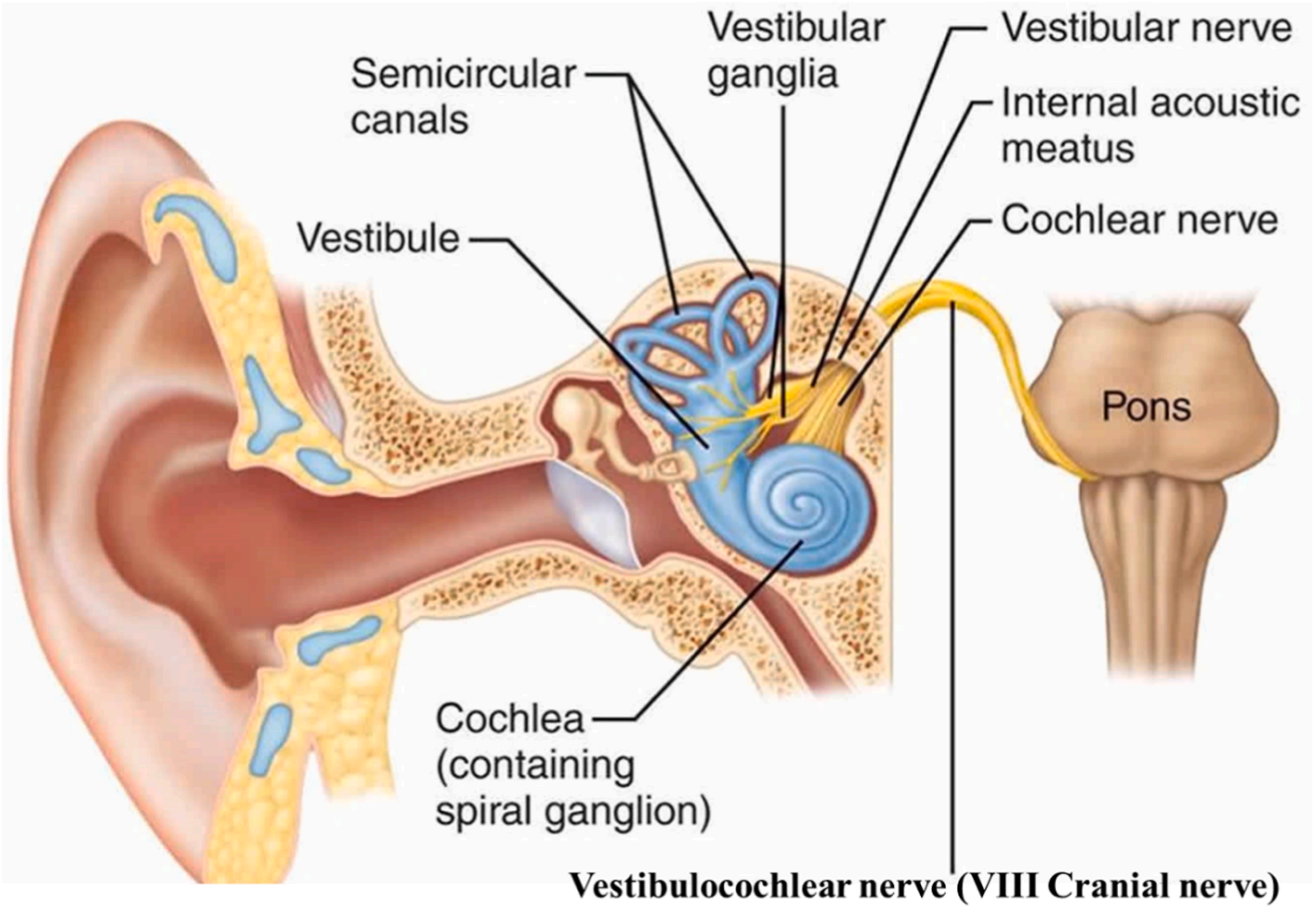
Details the anatomical structure of the vestibulocochlear nerve. The vestibular nerve controls the balance of the body, whereas the cochlear nerve plays a vital role in the sound processing system by maintaining close coordination with the brain and central nervous system.

The primary function of the vestibular system is to maintain coordinated communication between the vestibular apparatus (semicircular canals, saccule, utricle), ocular muscles, postural muscles, brainstem, and cerebral cortex by relaying motion and position.^15^^,^^16^ The peripheral vestibular apparatus consists of a bony and membranous labyrinth located within the temporal bone. Sensory neuroepithelium-based membranous labyrinth situated within the bony labyrinth under the suspended condition in the perilymph. Anatomically, the vestibular apparatus is made with 5 main components: an utricle, a saccule, and 3 semicircular canals (anterior, posterior, and lateral) oriented at right angles to each other. The utricle and saccule contain the macula, which consists of hair cells, while the semicircular canals contain crista ampullaris. Hair cells are covered with an otolithic membrane, and the surface contains otoconia (calcium carbonate crystals). Head position monitoring and the necessary response to movement under gravity depend on the mass of the otoconia. The hair cell stimulation results in depolarization, and an enhanced rate of calcium influx promotes an increased firing rate of afferent vestibular nerve fibers. The main functions of the utricle and saccule are to sense the positioning of the head in space by responding to horizontal and vertical acceleration with hair cells within the utricle and the saccule, respectively. On the other hand, angular acceleration or head rotation was sensed by the hair cells within the semicircular canal.^17^ The following Fig. 2 represents the anatomical structure of the vestibulocochlear nerve.

Transmission of auditory signals through the external and middle ear to reach the inner ear and then to the cochlear nuclei in the brainstem and primary auditory cortex in the temporal lobe is controlled by the cochlear nerve.^15^^,^^18^ The hollow middle ear cavity works as an impedance-matching filter to transfer low-impedance acoustic energy from the air to the high impedance of fluid in the inner ear. In terms of anatomy, the cochlea is a spiral, fluid-filled cavity in the bony auditory labyrinth containing the organ of Corti along its basilar membrane situated within the inner ear cavity. The 3 main parts of the cochlea are scala vestibuli, scala tympani, and scala media (cochlear duct), separated by the basilar membrane and the vestibular membrane, respectively. The scala vestibule and scala tympani contain endolymph, whereas the scala media contains perilymph. The cochlear nerve is made with cell bodies of bipolar neurons in the spiral (cochlear) ganglion, interlinked with the central nervous system and the organ of Corti. The spiral ganglion consists of the spiral canal of the modiolus and is made up of Type I and Type II neurons, which participate in peripheral as well as central processes and form the cochlear nerve. Anatomically, Type I neurons are larger and myelinated, and about 90% of the cochlear nerve contains them, whereas smaller-sized Type II neurons are nonmyelinated. The main function of Type I and Type II neurons is to project to the inner and outer hair cells of the organ of Corti, respectively.^19^, ^20^, ^21^, ^22^ Incident acoustic waves generate vibrations in the cochlea, introducing pressure waves in the scala vestibule in terms of vibration. The vestibular membrane vibration then transfers pressure waves from the perilymph to the endolymph of the scala media. The function of the basilar membrane is to generate vibration, which bends the stereocilia of the hair cell, resulting in depolarizing it. Based on the mechanical properties of the basilar membrane, it is revealed that sound of higher frequencies is mainly detected at the base of the cochlea, whereas lower frequencies are at the apex of it.^17^^,^^23^ Research revealed that diseases affect the sensory receptor organs of the vestibular and auditory systems more than the nerve itself.^2^ However, the discerning effect of TBI is not limited to the auditory sensory receptors only; it may also damage the nerve, promote pathological growths, and reduce cochlear fluid, which promotes the deterioration of auditory function.^2^^,^^24^^,^^25^ Therefore, TBI-associated vestibulocochlear nerve damage plays an important role in primary or secondary auditory dysfunction after TBI, which is discussed in the next section.

## 4 TBI-associated damage in the vestibulocochlear nerve

The latest research on TBI revealed that vestibulocochlear dysfunction is a common outcome in patients with acute TBI. A preliminary finding showed that adult patients with chronic moderate to severe TBI are prone to dizziness and imbalance due to vestibular impairment.^26^ Other findings demonstrated that dizziness is a somatic symptom and it may appear in chronic mild TBI.^27^ Studies revealed that peripheral vestibular dysfunction may arise due to inner ear damage caused by primary blast impact with or without mild TBI.^28^ Significantly, rates of vestibular dysfunction are similar in blast and non-blast TBI groups.^29^ Common complaints of vestibular dysfunction include dizziness, vertigo, nausea, fogginess, unsteady gait, and postural instability.^30^, ^31^, ^32^, ^33^ Vestibular dysfunction is well-documented in patients with persistent post-concussive symptoms after TBI.^30^^,^^34^, ^35^, ^36^, ^37^ The relationship between post-traumatic stress disorder and vestibular abnormality is a well-established fact without objective vestibular impairment.^38^^,^^39^ The patients suffering from vestibular-related dizziness were shown physical, emotional, and functional abnormalities, which led to a lack of work efficiency. Studies showed that dizziness leads to decreases in work efficiency and complete disability.^40^ Specifically, vestibular-associated dizziness can degrade the quality of life of the elderly by increasing the related risk factors, such as lack of attention and falls.^41^ Cognitive dysfunction may also appear due to dizziness caused by vestibular dysfunction.^42^ Recent research revealed a relationship between the vestibular system and cognition.^43^ Studies based on animal and human models with vestibular pathology indicated spatial orientation and memory deficits.^44^ Advanced literature indicated that the loss of vestibular function can cause long-term cognitive impairments, which may affect attention, memory, communication, and problems with executive function.^45^ The vestibular damage may alter the structural and functional activity of the brain's cortical regions, including the temporoparietal junction, parietal cortex, cingulate gyrus, posterior parietal cortex, hippocampus, and parahippocampal cortex, which are directly associated with vestibular input.^46^ The structural and functional changes of the vestibular-associated regions caused the upregulation of non-vestibular area networks, resulting in complications in cognitive and neural function.^47^ Literature showed that vestibular-related dizziness can cause cognitive dysfunction 4 times more than in normal individuals.^48^

A recent report demonstrated reduction in distortion product otoacoustic emissions, increased middle-ear muscle reflexes, extended auditory brainstem response (ABR) Wave I and ABR Wave III latencies for click and 4 kHz tone burst elicitors, higher middle latency responses (MLR) Na (first negative wave) and Nb (negative wave) amplitudes, low MLR Pb (positive wave) amplitudes, higher MLR Pa (first positive wave) latencies, and lower late latency responses N1 amplitudes for individuals with mild TBI. In addition, auditory evoked potentials promote thalamic and cortical dysfunction in individuals with mild TBI.^49^ The latest findings on a post-TBI human temporal bone pathology study demonstrated a remarkable loss in the cochlea in terms of loss in outer hair cells, upper basal, lower, and upper middle turns, a loss of spiral ganglion cells, and cochlear hemorrhage in the basal turns without any skull fracture.^50^ TBI-associated macrophages in the cochlea can cause progressive hearing loss.^51^ A mild TBI blast study on the chinchilla model showed that ABR and distortion product otoacoustic emission level shifts in blast animals and immunofluorescence images revealed the variation of auditory cortex damage in different intensities of blast injury.^52^ Another similar study on the chinchilla model indicated that blast exposure can cause dysfunctions in central and peripheral auditory although the response in central auditory is independent of damage-induced peripheral response. Auditory dysfunction was identified in blast-exposed chinchillas in terms of the higher value of ABR thresholds, lower ABR wave I amplitudes, and apoptosis of cells in the inferior colliculus.^53^ The direct impact of shockwave transmission in the entire region of the central nervous system to auditory pathways promotes dysfunction in the central auditory system,^54^ which recommends an option to utilize central auditory neurotransmitters for therapeutic targets to control the abnormalities. An interesting research finding predicted a relation of cochlear impairment as a specific biomarker in Parkinson's disease.^55^ Research findings showed that tinnitus development can be caused by blast-induced central auditory neurodegeneration regardless of peripheral cochlear damage.^56^ An assessment of blast-induced auditory damage on mice model presented severe, long-term hearing loss due to slow tympanic membrane healing or a change in the mechanical properties of healed tympanic membranes after rupture; loss of basal outer hair cells, synaptopathy, and balance deficits after the TBI incident.^57^ Blast-TBI-induced overpressure promotes an increase in protein expression within the auditory system to help gliosis. An innovative strategy to treat neuroprotectin D1 showed the potential to reduce or halt auditory microglial cell activation caused by blast-TBI.^58^ A preventive measure in blast-TBI showed a localized neuronal cytoskeletal elements degeneration in the cochlear nucleus, revealing that the damage resulted from shockwave transmission via the ear canal, which can be prevented by using proper ear protection devices against acute and chronic central auditory signal processing defects like tinnitus.^59^^,^^60^ A research finding indicated that cardiovascular risk factors like heart rate variability, a common sequel of TBI, were a significant predictor for cochlear impairment.^61^ Different types of TBI, such as closed head injury, focal injury, diffuse injury, and blast injury, may affect the auditory functions differently and result in injury-specific abnormalities. The following table demonstrates clinical studies on TBI-specific hearing dysfunction (Table 1).

**Table 1:** Tramatic brain injury (TBI) impacts on hearing dysfunction.

TBI type	Study model	Key findings	Reference
Maxillofacial trauma	Soldier's	Hearing impairment	62
Brain concussion	Patients	Hearing loss, conductive deafness, tear of the eardrum found	63
Mild TBI	58-year-old woman	Pure-tone detection demonstrated auditory processing deficits	64
Skull fracture and intracranial injury	Adult patient	Demonstrated enhanced risk of long-term hearing loss	65
Blast exposure	Military	Represent peripheral hearing loss, central auditory processing deficits, vestibular impairment, and tinnitus	66
Head injury	Children	Results revealed conductive hearing loss of 32% and 16% of high-frequency sensorineural hearing loss incidence	67
Closed head injury	Adult patients	Progressive hearing loss was revealed after the closed head injury	68
Brain Injury	10 years follow-up study of adult patients	Significant hearing impairment was present in group and one-to-one conversations	69
Closed head injury	30-year-old woman	Finding showed auditory dysfunction 13 years after the incident	70
Minor head injury	Adult patients	Results showed hearing loss	71
Brain injury	Patient suffered from TBI 19 months to 27 years ago	Findings included tinnitus, vestibular dysfunction, abnormal facial sensory symptoms, and intolerance to loud/sudden noises	72
Brain injury	Mild, moderate, and severe TBI patients	Demonstrated a proportional relationship between high-frequency sensory-neural hearing loss and injury severity	73
Brain injury	Patients with mild TBI in the last 3 to 18 months	Post mild TBI change of subcortical neural encoding of auditory information was observed, which may alter auditory processing	74

## 5 Health impact associated with auditory impairment

**Fig. 3:**
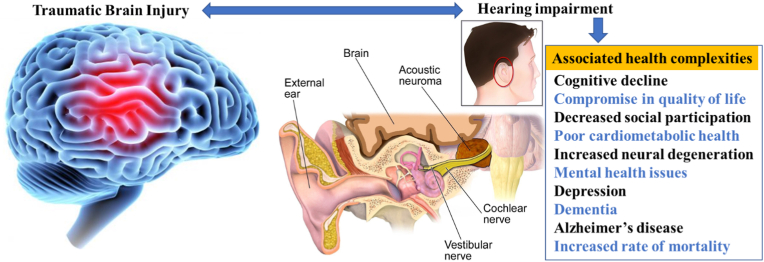
Impact of traumatic brain injury-associated hearing loss on other common health problems.

TBI-associated hearing impairment promotes multiple other health issues, like cognitive decline in older adults, decreased social participation, increased chance of dementia, psychiatric problems, depressive behavior, etc. The latest findings revealed that hearing intervention can affect long-term cognitive change in older adults and enhance cognitive decline over time.^75^ There is a relationship between hearing problems and frailty in later life.^76^ Evidence showed that hearing loss is related to poor cardiometabolic health profiles, which led to microvascular disruptions and increased neural degeneration. The same research indicated the relation between impaired auditory function and increased risk of falling, which is also a potential risk factor for cognitive decline and dementia.^77^ A study on amateur and professional musicians demonstrated hearing loss as one of the causes of mental health issues.^78^ Another finding presented the link between hearing loss and lack of social participation with depressive behaviors.^79^ Hearing loss may affect quality of life, and treatment of hearing loss significantly improves healthy behavior.^80^ Rapid cognitive decline was assessed with hearing loss and bilateral vestibulopathy.^81^ Less physical activity is associated with impaired hearing function, which is an important health parameter to maintain good health.^82^ A study concluded that hearing dysfunction is a direct cause to increase the risk of depression among aged adults, which can be reduced by the prevention and treatment of hearing problems.^83^ Advanced studies on audiology showed that premature ovarian insufficiency is associated with hearing loss, and early intervention by audiologists can improve the condition.^84^ A retrospective study indicated that amyloid pathology is associated with hearing function in younger-old individuals only, and they are more prone to decline in language, whereas oldest-old individuals are affected with a decline in memory, global cognition, and language due to the hearing impairment. The hearing loss-associated effect on memory and global cognition in the oldest-old individuals is caused by the hippocampus and nucleus accumbens.^85^ Hearing loss is severely impacted in cancer survivors. Reports presented that survivors over 6 years from their cancer and audiometrically confirmed hearing loss showed clinically significant levels of depression, anxiety, fatigue, energy decrements, insomnia, cognitive dysfunction, and pain.^86^ Research revealed that mild cognitive impairment with hearing loss reduced functional communication between the bilateral insular and anterior sections of the cingulate cortex and degraded fractional anisotropy in the bilateral fornix, major, and tapetum corpus callosum forceps, left parahippocampal cingulum, and left superior thalamic radiation.^87^ Studies indicated the link between social stigma and auditory dysfunction, which is highly related to withdrawal, social segregation, and negative self-perception.^88^ A study on unilateral hearing loss in children showed a higher level of fatigue and degradation in quality of life than in children with no hearing problem.^89^ A pilot study on hearing impact to cope with the social challenges indicated the negative sequelae on social and emotional experiences, identity impact, and emotional distress. At the same time, some participants showed a lack of coping ability, and others suffered from social isolation and avoidance.^90^ The adjacent Fig. 3 demonstrates the health adversities caused by hearing dysfunction.

A relationship between hearing loss and mortality suggests that there is a higher rate of mortality in patients suffering from chronic auditory dysfunction due to infection, neoplasm, trauma, metabolic, mental, circulatory, respiratory, and digestive diseases than in normal people without any hearing problem.^91^ The increasing research interest in this field may reveal more correlation of hearing impairment effect with other health complexities in the future.

## 6 Conclusion

The versatile health impact of TBI incidents is affecting the auditory system just after the accident or at any time after the scene. Slight damage or injury to the vestibulocochlear nerve may cause lifelong hearing impairment with or without medical intervention. TBI-induced secondary hearing abnormality brings multiple other health complexities, such as psycho-social abnormality, cognitive impairment, behavioral problems, dementia, and many more. Hearing impairment in childhood may degrade the quality of life for the rest of one's life. Hearing dysfunction is associated with increased accumulation of β-amyloid, which increases the risk of Alzheimer's disease in aged people with auditory dysfunction. Cardiac dysfunction is considered a significant health issue that is associated with hearing abnormalities and TBI. The venomous tentacles of TBI-associated impairment in normal hearing may cause multiple other health issues, which increase the mortality rate. Therefore, early intervention by the audiologist for TBI patients to follow up on the audiological dysfunction is an advanced strategy to tackle and minimize the worst health outcomes. Specifically, a standard protocol should be initiated in the emergency trauma care department to screen post-TBI auditory functions such as audiometry, tympanometry, otoacoustic emission, and ABR test to assess frequency responses, eardrum movement, cochlear function, and the brain's response to sounds, respectively, for understanding external, middle, and inner ear problems to recommend the requirement of necessary audiological intervention. This work also highlighted the post-TBI multi-dimensional health abnormalities that may be caused by hearing problems, which makes this unique.

## CRediT authorship contribution statement

**Fazle Kibria:** Writing – original draft, Conceptualization, Writing – review & editing. **Sudip Kumar Das:** Writing – review & editing.

## Ethical statement

Not applicable.

## Funding

Nil.

## Declaration of competing interest

The authors declare that they have no conflict of interest.
